# A Randomized Pilot Trial of Contingency-management Intervention for Patients in during Methadone Maintenance Treatment, Cash vs. Vouchers

**Published:** 2018-04

**Authors:** Bijan PIRNIA, Ali SOLEIMANI, Hamid Reza TAHERINAKHOST, Kambiz PIRNIA, Parastoo MALEKANMEHR, Alireza ZAHIRODDIN

**Affiliations:** 1. Dept. of Psychology, Faculty of Humanities, University of Science and Culture, Tehran, Iran; 2. Behavioral Sciences Research Center, Shahid Beheshti University of Medical Sciences, Tehran, Iran; 3. Iranian National Center for Addiction Studies, Tehran University of Medical Sciences, Tehran, Iran; 4. Technical Assistant in Bijan Center for Substance Abuse Treatment, Tehran, Iran; 5. Dept. of Psychology, Branch of Hamedan, Islamic Azad University, Hamadan, Iran; 6. Dept. of Psychiatry, Behavior Research Center, Shahid Beheshti University of Medical Sciences, Tehran, Iran

## Dear Editor-in-Chief

Cocaine usage is common among patients under the treatment of methadone maintainer. Using stimulants, by affecting HPA axis and the role of anxiety, facilitates the process of relapse in the period of abstinence. Therefore, it is worthy of clinical attention (1).

Contingency management (CM) has recently shown efficacy in promoting abstinence in the realm of drug abuse (2). CM shapes behavior in form of using secondary positive boosters such as coupons, goods and services. The efficacy of contingency management Approach for avoidance of wide range of drugs including stimulants has been shown (2, 3). This three-group randomized trial compared the efficacy of cash-based reinforcement therapy (CBRT, n=25) to voucher-based reinforcement therapy (VBRT, n=25) and a non-CM condition (n=25) on opiates and cocaine during Methadone Maintenance Treatment. From Sep 15 to Nov 15, 2014, from Tehran cocaine use in methadone outpatient in Bijan Center for Substance Abuse Treatment, Tehran, Iran 75 individuals (Age range=18–60 yr; SD=3.18) were selected by Respondent-Driven Sampling (RDS) and were assigned using Microsoft excel.

The informed consent was obtained from the participants and the whole process was carried out based on the latest version of the declaration of Helsinki (Clinical Trial Registration Code: TCTR20180329002).

Eligibility criteria for initial enrollment included: 1) Male patients; 2) fulfilling DSM-IV diagnostic criteria for cocaine and opioid dependence; 3) eligible for methadone maintenance. Gift card (coupon) was used for buying from supermarkets, bus pass, toiletries or any five large prizes. Participants in the Cash-Based CM Condition identical 12-week escalating schedule of reinforcement, except that the contingencies were provided in cash rather than vouchers (Cash-based incentives worth $0, $20, $40, and $80). The case manager’s estimated total annual compensation was $41332 ($19.87 per hour). Urine specimens were analyzed by enzyme-multiplied immunoassay technique (EMIT) system with cutoff concentrations for positive set at 300 ng/ml. The data were analyzed by generalized estimating equations (GEE) models by IBM SPSS Statistics Version 20 (IBM Corp., Armonk, NY, USA).

The Primary outcome showed these two treatments did not have a significant effect on increasing negative urine test of the stimulant, The mean of (95% of confidence interval) number of negative cocaine urine tests was 13.6 (12.0–15.2) in CBRT group, 14.3 (12.3–16.2) in VBRT group and 15.1 (13.5–16.9) in non-CM condition (*P* = 0.07). But, Secondary outcome showed both CBRT and VBRT group have significant effects on reducing the craving than the non-CM group (all *p*’s < .05).

These findings are in contrast with the results of studies by Festinger et al. (4) that showed both vouchers-based and cash-based treatments create significant difference in therapeutic responsiveness with the aim of decreasing cocaine usage. The difference in the number of the participants is one of the differences of the present study with the studies by Festinger et al. (4) that can be effective on significance of the mentioned hypothesis. The other difference is for mean of the cost of given cash in the present study (mean of 230 dollars) that was lower than the other study.

Consistent with the previous findings (5, 6) the results of this study supports the safety of giving cash to participants and it facilitates the process of doing the research and solves the problems due to giving vouchers. Hypothesis was that giving cash can be more effective than giving vouchers. Several factors have limited the effectiveness of giving cash in the present study. The first thing is that because of security and legal issues, giving cash was not done immediately but was done in a set and late at night through paying on-line. Giving money at late time can limit the motivation to create risky behaviors such as providing drug or planning for usage. However, what can reduce the effectiveness of such a reward is creating the interval of about 8 hours that reduces the efficacy of cash. Finally, Because of some limitations, the sample of the present study was consisted of just male methadone users, it can be attended in future studies.
